# Selective Recovery of Gold from Electronic Waste by New Efficient Type of Sorbent

**DOI:** 10.3390/ma16030924

**Published:** 2023-01-18

**Authors:** Grzegorz Wójcik, Magdalena Górska-Parat, Zbigniew Hubicki, Karolina Zinkowska

**Affiliations:** Department of Inorganic Chemistry, Institute of Chemical Sciences, Faculty of Chemistry, Maria Curie-Skłodowska University, Maria Curie-Skłodowska Sq. 2, 20-031 Lublin, Poland

**Keywords:** precious metals, impregnated resin, secondary sources, waste electrical and electronic equipment (WEEE)

## Abstract

Modular connectors are applied by computer users, and they can be metallic secondary sources containing metals such as gold and copper. Because gold is a micro-component, the solution obtained after the pin digestion contains a low concentration of gold(III) ions, and efficient and selective sorbent should be used for gold(III) ion recovery. The selective removal of small amounts of gold(III) from 0.001–6 M hydrochloric acid solutions using pure and solvent-impregnated macroporous polystyrene crosslinked with divinylbenzene sorbents (Purolite MN 202 and Cyanex 272) is presented. Gold(III) ions were recovered effectively from the chloride solution after the digestion of the modular connector RJ 45 (8P8C) using Purolite MN 202 after the impregnation process. The dependence of the recovery percentage (R%) of gold(III) on the contact time was determined. The highest value of gold(III) ion sorption capacity (259.45 mg·g^−1^) was obtained in 0.001 M HCl for Purolite MN202 after the Cyanex 272 impregnation. The results can be applied to gold recovery from e-waste. The presented method of gold recovery does not generate nitrogen oxides and does not require the use of cyanides.

## 1. Introduction

In addition to being prized for millennia, recently precious metals have been employed in a great number of technical applications due to their unique physical and chemical properties. The main applications of platinum and palladium are in electronic and catalytic processes in the chemical industry, and the number of chemical processes based on the palladium or platinum-catalysed reactions is still growing. These include hydrogen synthesis and catalytic conversion. Gold has been used for a long time as currency as well as in the electronic, medical, and jewellery industries [1].

In recent years, scraps containing gold are more sought after than ever before as a source of income. They can originate from different sources such as jewellery and spent catalysts as well as waste electrical and electronic equipment (WEEE), called “E-waste”, which is considered to be one of the fastest growing waste streams in Europe [2,3,4]. It is estimated that the amount of WEEE generated in 2023 will reach 61.3 million tonnes [5].

Prices for noble metals fluctuate drastically. The price of gold in December 2022 was USD 1750 per troy ounce, and in 2023 it may rise to USD 3000. High prices as well as scarcity contribute to the search for new methods of precious metal recovery, as traditional methods of recovery require aggressive reagents that exert a negative impact on the natural environment [6].

Numerous techniques have been described for gold recovery from e-waste: pyrometallurgical processing, hydrometallurgical processing, or bio-hydrometallurgical processing [2,7].

Gold containing e-waste should be digested to extract gold into a solution. Both ion-exchange and solvent extraction (SX) methods can be used for recovery and purification of gold(III) ions from solution. However, commonly used techniques for separation and recovery of precious metal ions from concentrated solutions are not effective at lower concentrations. The ion-exchange resins are more effective than the solvent extraction processes at lower concentrations of gold(III) ions [6]. Gold(III) ions can be removed by resins containing functional groups with S donor atoms. The maximum sorption capacity, determined to be approximately 22 mg·g^−1^ of sorbent, was obtained at the initial gold concentration of 150 mg·dm^−3^ [8]. Studies of gold(III) ion sorption from hydrochloric acid solutions on 2,2′-thiobisethanol dimethacrylate/ethylene glycol dimethacrylate copolymer were carried out. The correlation between the concentration of HCl (0.0001–4 M) and the sorption efficiency (370–140 mg·g^−1^) was observed [9]. Gold(III) ions can exist in cyanide solution. Activated carbon (NORIT GAC 1240), strong base (Purogold A194), and weak base (Purogold S992) resins were tested as the adsorbents. The activated carbon was found to be the best adsorbent as it achieved the largest gold loading [10]. Thiourea-modified chitosan-imprinted resin (IM-TUCS) was tested for gold(III) ion sorption. Protonation of the amino group on the resin at low pH value promoted Au(III) adsorption; O, N, and S in the C–OH, C=S, and C-NH_2_ groups contained in the IM-TUCS coordinated with Au(III) ions [11].

In the absence of impregnation, the resins are not selective enough to remove the desired metal ions from large amounts of the accompanying metals. The impregnated resins are selective for metal ions through the functional groups, which are immobilized in the resin matrix through the chemical bonds or the physical adsorption [12]. The solvent impregnated resins (SIRs) are usually prepared by immobilization of a solvent extraction reagent within the polymer matrix through physical contact. SIRs are not only simple to prepare but also can contain a variety of selective liquid–liquid extractants. SIRs represent a combination of ion-exchange and extraction processes [13]; they lose an extractant gradually in long-term use, but increased stability can be achieved by its coating with crosslinked water-insoluble polymers [14]. Cyanex 272 is a very common extractant suitable for extraction and separation of zinc(II), nickel(II), and cobalt(II) [15] as well as the extraction of lanthanum(III) [16] and uranium(VI) [17]. Cyanex 272 was used to impregnate such materials as silica in the zinc sorption [18] and the synthetic resins for the rare earth (III) extraction [19], Amberlite XAD-2 for Zn(II), Cu(II), and Cd(II) removal [20]. The modular connectors contain mainly copper, whereas gold is in very small amounts. Therefore, in the solution after the leaching, gold will be a micro-component. Taking these assumptions into account, an attempt was made to obtain a selective sorbent based on the Purolite MN 202 resin and the Cyanex 272 extractant. Purolite NM 202 was not examined for gold removal according to the Web of Science database. Purolite NM 202 was chosen because this is a macroporous resin that is easy to use for impregnation. Moreover, the macroporous structure offers fast kinetics of gold(III) ion sorption and is stable in hydrochloric acid solution. The main goal of this paper was the investigation of the applicability of Purolite NM 202 for gold(III) ion removal from solution after leaching electronic wastes such as modular connectors RJ 45 (8P8C). In addition to the preparation of the impregnated sorbent, the aim of the paper was to test the possibility of gold recovery from the spent connectors using a dissolution method that does not generate nitrogen oxides. The presented method for recovery of gold does not require the use of a toxic cyanide solution.

## 2. Materials and Methods

### 2.1. Materials

Purolite MN 202 was produced by the Purolite Company, Great Britain. Cyanex 272 was produced by Cytec Canada Inc. We obtained 36% HCl from POCh, Poland, and 30% H_2_O_2_ and acetone from Stanlab, Poland, which were all reagent grade. Sodium hydroxide produced by Chempur, Poland, was analytically pure. Demineralized water was prepared by the Polwater system DL2-150, Poland. The gold(III) 1000 μg·cm^−3^ reference solution was produced by ROMIL. Modular connectors RJ 45 (8P8C) were obtained from spent computer connectors.

### 2.2. Characteristics of Resin

**Figure 1:**
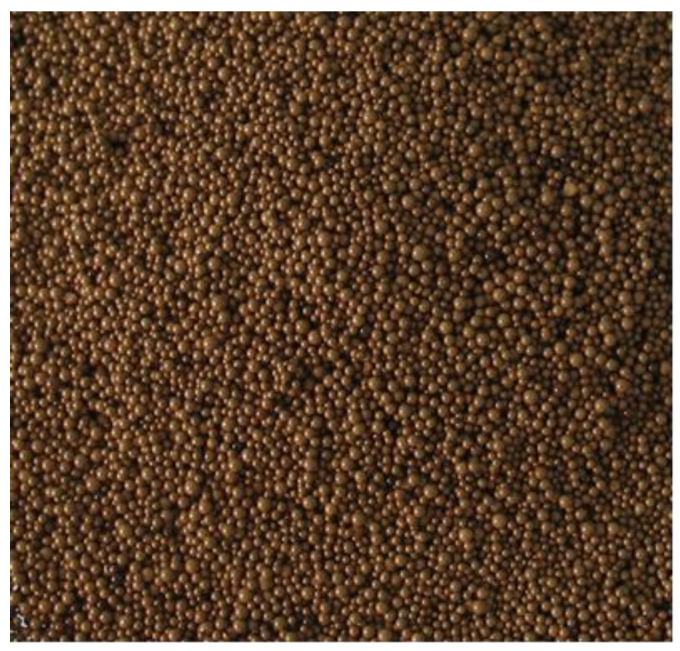
Purolite MN 202.

Purolite MN 202 (Figure 1) is an unfunctionalized macroporous polystyrene crosslinked with divinylbenzene. Its surface area is >700 m^2^·g^−1^, the pore volume is 1–1.1 cm^3^·g^−1^, and the mean particle size range is 0.45–0.62 mm.

### 2.3. Characteristics of Extractant

**Figure 2:**
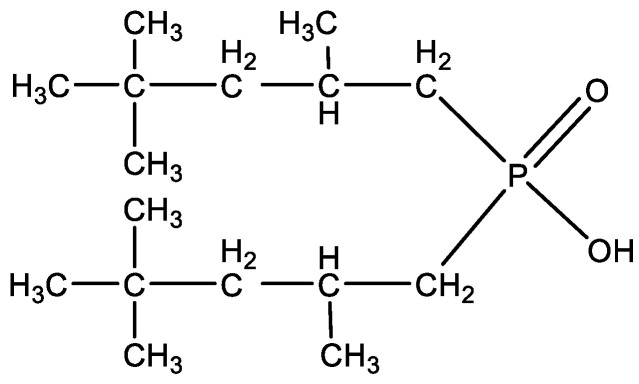
Structure of Cyanex 272.

Cyanex 272 (CAS No. 83411-71-6) is bis(2,4,4-trimethylpentyl)phosphinic acid (Figure 2). It is a colourless to light amber liquid with the solubility of 16 μg·cm^−3^ at pH 2.6 and 38 μg·cm^−3^ at pH 3.7 in distilled water. The flash point is over 381 K.

### 2.4. Methods and Analysis

#### 2.4.1. Preparation of Pure Resin

The Purolite MN 202 resin was washed three times with acetone to remove any impurities then rinsed thoroughly with the deionized water and air-dried at 298 K.

#### 2.4.2. Preparation of the Impregnated Resin

A 15 g sample of resin prepared according to Section 2.4.1 was mixed with 6 g of Cyanex 272 (ratio Cyanex 272 g: Purolite MN 202 g—0.4:1) and stirred for 4 h. The mixture of impregnated resin and solution was separated by filtration and the solid was washed with distilled water to remove the impregnation mixture and then dried at 298 K.

##### Determination of Cyanex 272 Concentration in Purolite MN 202

Determination of Cyanex 272 concentration in Purolite MN 202 was performed by the titration of 0.104 g Cyanex 272 in the anhydrous medium (25 cm^3^ acetone) using the 0.1 M NaOH solution with phenolphthalein as an indicator. The end point for the blank titration occurred at 3.5 cm^3^ of NaOH. A 1.004 g sample of Purolite MN 202 impregnated with Cyanex 272 in the anhydrous medium (25 cm^3^ acetone) was titrated with the 0.1 M NaOH solution using phenolphthalein as an indicator.

##### FTIR Studies

A Perkin-Elmer 1725X Fourier transform infrared (FTIR) spectrometer (Waltham, MA, USA) was used to identify molecular structures present in the KBr pellets containing the samples. The FTIR spectra were recorded for both Purolite MN 202 and Purolite MN 202 after the impregnation by Cyanex 272.

##### Surface Area

The samples were placed in a Micromeritics Accelerated Surface Area and Porosimetry ASAP 2405 instrument and degassed at 293 K. The surface area, total pore volume, and average pore diameter were determined for both Purolite MN 202 and Purolite MN 202 after the Cyanex 272 impregnation process.

#### 2.4.3. Preparation of Metal Solutions

Hydrochloric acid solutions (0.001–6 M HCl) containing 100 μg·cm^−3^ gold(III) ions were prepared before the experiment. The real solution was prepared by leaching pins in the hydrochloric acid–hydrogen peroxide system.

#### 2.4.4. Adsorption Studies

A 0.25 g portion of dry pure resin was placed in a 100 cm^3^ Erlenmeyer flask closed with a ground-glass stopper. A 25 cm^3^ aliquot of metal ion was added, and the flask was shaken for 1–360 min at 293 K using type 385 S laboratory shaker (produced in Poland). The solution was filtered and the gold(III) ion concentrations were determined using atomic absorption spectrometry (Varian 240). The same procedure was employed for the impregnated resin. The gold(III) ion concentrations in the solution after leaching pins were determined using the standard addition method to eliminate matrix interferences.

##### Desorption Studies

For the desorption studies, 0.1 g of sorbent (before and after the impregnation) was loaded with gold(III) using 10 cm^3^ of 100 mg·dm^−3^ gold(III) solution in 3 M HCl, and an agitation period of 24 h was applied. Five percent thiourea in the 0.1 M HCl solution was used as the desorption agent. The gold(III) ion concentrations in the solutions were determined by the AAS method.

#### 2.4.5. SEM

The scanning electron microscopy (SEM) measurements were conducted by means of the Quanta 3D FEG scanning electron microscope produced by the FEI Company (Lincoln, NE, USA).

## 3. Results and Discussion

### 3.1. Sorption Studies of Au(III) on Purolite MN 202

**Figure 3:**
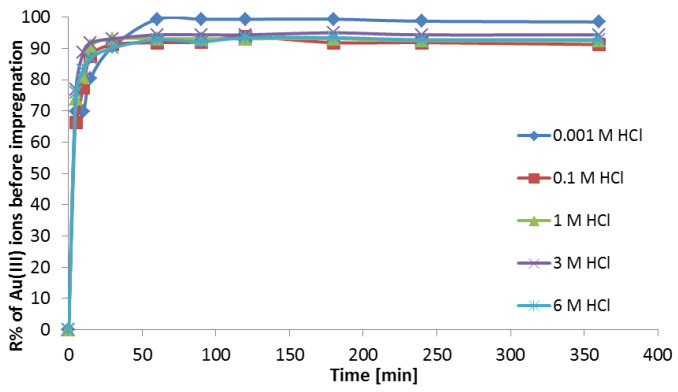
Influence of hydrochloric acid concentration and phase contact time on the removal of Au(III) ions (100 μg·cm^−3^) from the aqueous solution using Purolite MN 202.

The recovery percentage (R%) of gold(III) was calculated using [21]:

(1) $$R\%=\frac{C}{C_{0}}\times 100\%$$

in which *C* is the concentration of adsorbed gold(III) ions calculated from the difference in the solution concentration before and after the sorption process, and *C*_0_ is the initial concentration of gold(III) ions. The influence of the hydrochloric acid concentration on the ion sorption on Purolite MN 202 is illustrated in Figure 3.

The sorption of gold(III) ions on Purolite MN 202 is presented in Figure 3. Hydrochloric acid affects the gold(III) ion sorption. The value of R% is 99.3% in 0.001 M HCl. In the hydrochloric concentration range 0.1–6 M, the R% values are 91.25–94.37%. The recovery percentages R% are higher in 0.001 M than in the range 0.1–6 M HCl for the gold(III) ions.

### 3.2. Extraction Studies of Au(III) Using Cyanex 272 in Toluene

**Figure 4:**
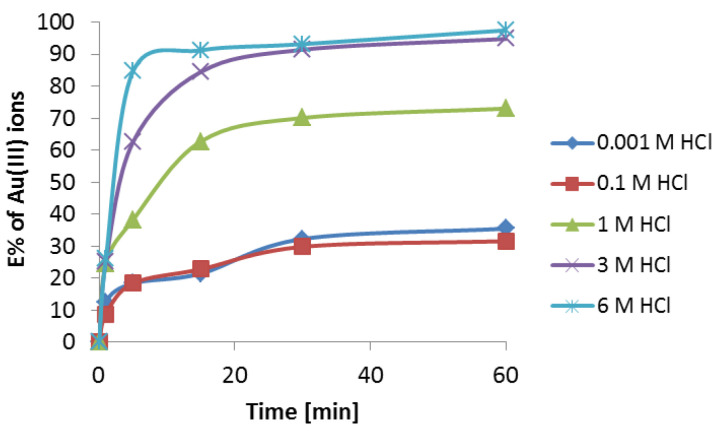
Influence of hydrochloric acid concentration and phase contact time on the extraction of Au(III) ions (100 μg·cm^−3^). The phases were composed of the 25 cm^3^ aqueous phase in contact with the 25 cm^3^ organic phase (5% *v*/*v* Cyanex 272 in toluene).

The extraction percentage (*E*%) for gold(III) were calculated from:

(2) $$E\%=\frac{C}{C_{0}}\times 100\%$$

in which *C* is the gold(III) ion concentration calculated from the difference in concentration before and after the extraction in the aqueous phase, and *C*_0_ is the initial concentration of gold(III) ions. The influence of HCl concentration on the gold(III) extraction using Cyanex 272 is presented in Figure 4.

Extraction of gold(III) was dependent on the HCl concentration. Extraction of gold(III) ions was the most effective in the range of 3–6 M HCl concentration, with the *E*% being approximately 90%. In 1 M HCl, E% was 70%. The least effective conditions were in the 0.001 to 0.1 M HCl concentration range, with E% values of approximately 30%. The dependence of *E*% on the HCl concentration can be written as follows: 6 M > 3 M > 1 M > 0.1 M > 0.001 M. The strong HCl concentration dependence suggests that the extraction of gold ions proceeds through the coordination process. The oxygen atom in the Cyanex 272 molecule is a donor of electron pair, whereas the gold(III) ion as a transition metal is able to accept the electron.

### 3.3. Sorption Studies of Au(III) on Purolite MN 202 Impregnated with Cyanex 272

#### 3.3.1. Determination of Cyanex 272 Concentration in Purolite MN 202

The titration experiment was conducted to estimate the amount of Cyanex 272 in Purolite MN 202. The titration endpoint occurred after the addition of 3.54 cm^3^ of 0.1 M NaOH. The concentration of Cyanex 272 per 1 g Purolite MN 202 was estimated to be 0.105 g·g^−1^.

#### 3.3.2. FTIR

**Figure 5:**
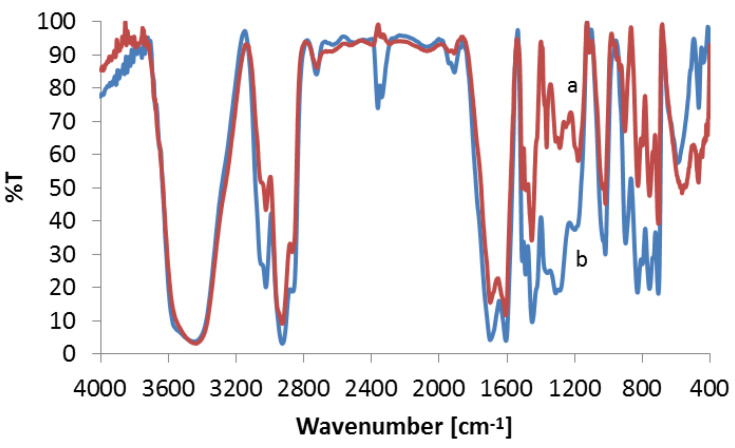
FTIR spectra of Purolite MN 202 (a) and Purolite MN 202 impregnated with Cyanex 272 (b).

The additional evidence for the impregnation of Purolite MN 202 was obtained using FTIR spectroscopy. The transmission mode bands observed in Purolite MN 202 and Purolite MN 202 treated with Cyanex 272 are presented in Figure 5. The major modifications visible in the spectrum of the treated material include strong bands at 1364 cm^−1^ attributed to the P=O stretching vibrations and at 540 cm^−1^ P-O.

The broad band at 3458 cm^−1^ is related to OH from water. The alkenyl C-H band occurs at 3024 cm^−1^. The aromatic ring C=C stretching vibrations occur at 1604 cm^−1^. The bands at 902 cm^−1^ and 762 cm^−1^ correspond to the aromatic =C-H deformation vibrations for the substituted benzene ring.

#### 3.3.3. Surface Area

To identify the changes for Purolite MN 202 before and after the impregnation, the sorption studies employing nitrogen were carried out. The surface area of Purolite MN 202 was initially 818 m^2^·g^−1^, decreasing to 566 m^2^·g^−1^ after the impregnation. The total volume of pores smaller than 968.8 Å was 0.547 cm^3^·g^−1^, decreasing to 0.428 cm^3^·g^−1^ after the impregnation. The surface area and the total pore volume decreased because of the Cyanex 272 location in the sorbent pores.

The average pore size of the sorbent before the impregnation was 21.25 Å. This increased to 23.98 Å after the impregnation by Cyanex 272, indicating that the molecules of Cyanex 272 were located in the sorbent micropores.

#### 3.3.4. Adsorption Investigations

**Figure 6:**
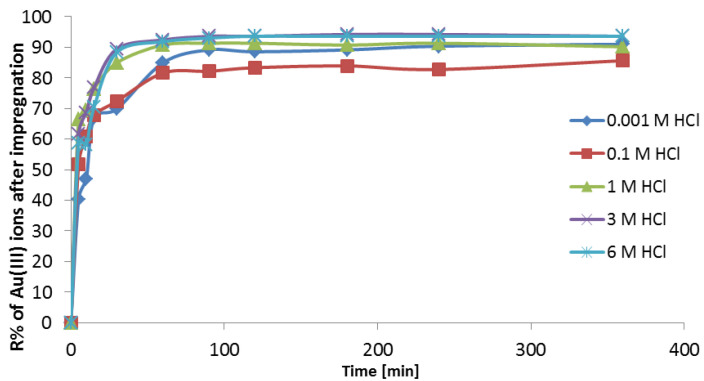
Influence of hydrochloric acid concentration and phase contact time on the removal of Au(III) ions (100 µg·cm^−3^) from the aqueous solution using Purolite MN 202 impregnated with Cyanex 272.

The influence of hydrochloric acid concentration on the gold(III) ion sorption on Purolite MN 202 impregnated with Cyanex 272 is depicted in Figure 6. The impregnated sorbent was characterized by good sorption of Au(III) ions, which was dependent on the HCl concentration. The values of R% increased slightly with increasing HCl concentration. The highest sorption of gold(III) ions was observed in the HCl concentration range 3–6 M (R%—94–95%). The maximal removal of gold(III) ions in the hydrochloric acid concentration range 3–6 M is similar to the results obtained in the extraction process. After the impregnation, the presence of Cyanex 272 on the Purolite MN 202 surface affects the mechanism of gold(III) ion sorption. The high sorption values of R% in the 3–6 M HCl concentration range are related to the coordination process of gold(III) ions by the donor atoms such as oxygen in the Cyanex 272 structure.

### 3.4. Kinetic Parameters

The sorption mechanism of Au(III) ions on pure and Cyanex 272-impregnated Purolite MN 202 was modelled assuming the pseudo-second-order kinetics of the form [22,23,24]:

(3) $$\frac{dq_{t}}{dt}=k_{2}{\left(q_{2}-q_{t}\right)}^{2}$$

in which *q*_2_ is the amount of Au(III) ions sorbed at the equilibrium in mg·g^−1^, *q_t_* is the amount of metal ions sorbed at the time *t* in mg·g^−1^, and *k*_2_ is the pseudo-second-order equilibrium rate constant (g·(mg·min)^−1^). Integrating Equation (3) with the boundary conditions *q_t_* = 0 at *t* = 0 and *q_t_* = *q_t_* when *t* = *t* yields:

(4) $$\frac{1}{\left(q_{2}-q_{t}\right)}=\frac{1}{q_{2}}+k_{2}t$$

Equation (4) can be written in the linear form as Equation (5):

(5) $$\frac{t}{q_{t}}=\frac{1}{k_{2}q_{2}^{2}}+\frac{1}{q_{2}}t$$

and the initial sorption rate *h* (mg·(g·min)^−1^) is described by:

(6) $$h=k_{2}q_{2}^{2}$$

The kinetic parameters were calculated using the Microsoft Excel 2010 spreadsheet program.

The pseudo-second-order kinetic parameters obtained using Purolite MN 202 before and after the impregnation with Cyanex 272 for various HCl concentrations are presented in Table 1.

After the impregnation, the values for *k*_2_ and *h* are smaller for gold(III) ions than before the impregnation process, possibly because the presence of Cyanex 272 exerts changes on the physicochemical properties of Purolite MN 202. In addition to changing the physical sorbent properties such as surface area, pore volume, and volume diameter, the presence of Cyanex 272 affects chemical properties, as well. The changes in the kinetic parameters after the sorbent impregnation by the extractant indicate that the sorption mechanism is different from that before the impregnation process. The molecules of Cyanex 272 present in the polymer can react with gold(III) ions by the coordination reaction.

### 3.5. Adsorption Isotherms Models

The Langmuir and Freundlich models are two of the most widely applied adsorption models [25]. Both were employed for characterization of gold(III) ion adsorption on Purolite MN 202 before and after the impregnation process. Linear regression was used to determine the most fitted isotherm. The linearized form of the Langmuir model can be written as:

(7) $$\frac{C_{e}}{q_{e}}=\frac{1}{bQ_{0}}+\frac{C_{e}}{Q_{0}}$$

where *q_e_* is the amount of gold(III) ions in the adsorbent (mg·g^−1^); *C_e_* is the equilibrium concentration of gold(III) ions (mg·dm^−3^); *b* is the Langmuir isotherm constant (dm^3^·g^−1^); and *Q*_0_ is the maximum monolayer coverage capacity (mg·g^−1^).

Further analysis of the Langmuir equation can be made based on the dimensionless equilibrium parameter, *R_L_*, also known as the separation factor.

(8) $$R_{L}=\frac{1}{1+bC_{0}}$$

where *C*_0_ is the initial concentration of gold(III) ions solution (mg·dm^−3^).

The Freundlich isotherm was calculated according to Equation (7):

(9) $$logq_{e}=logK_{F}+\frac{1}{n}logC_{e}$$

where *q_e_* is the amount of gold(III) ions in the adsorbent (mg·g^−1^); *K_F_* is the characteristic constant related to the adsorption capacity (dm^3^·g^−1^); *n* is the adsorption intensity; and *C_e_* is the equilibrium concentration of gold(III) ions (mg·dm^−3^).

The results of the calculated parameters using the Freundlich and Langmuir models are given in Table 2 for both impregnated and non-impregnated resins.

The adsorption capacity for the gold(III) ions is higher for the impregnated resin than for the non-impregnated one. The high adsorption capacity is achieved by the impregnation process. The presence of bis(2,4,4-trimethylpentyl)phosphinic acid in the Purolite NM 202 structure causes the impregnated resin to work like an ion-exchanger. The highest adsorption capacities are obtained in the HCl concentration range from 0.001 to 0.1 M HCl. This phenomenon can be explained by the fact that the reduction of gold ions is higher at lower hydrochloric acid concentration. The reduction process is fundamental for the sorption on the non-impregnated Purolite MN 202. Moreover, the sorption capacity increases insignificantly in the concentration range from 1 M to 6 M for Purolite MN 202 impregnated by Cyanex 272. This fact can be related to the coordination of gold(III) ions by the extractant oxygen atoms. In strong acidic media, 3–6 M HCl extractant molecules can be protonated and the anionic chloride complexes of gold(III) interact with the positively charged extractant molecules according to the reaction:

[EH^+^ + Cl^−^]_org_ + AuCl_4_^−^ = [EH^+^ +AuCl_4_^−^]_org_ + Cl^−^ (10)

where EH^+^ are the protonated molecules of Cyanex 272.

The overall Langmuir isotherm model had the higher regression coefficient (R^2^) compared with that of the Freundlich isotherm in the hydrochloric concentration range from 0.1 to 6 M. The *R_L_* values indicate favourable adsorption, as it is in the range 0 < *R_L_* < 1. The calculated *R_L_* values increase with the increasing hydrochloric acid concentration and reach the values from 0.118 in 0.001 HCl to 0.357 in 6 M HCl. The calculated values are smaller than 1 and indicate favourable adsorption of gold(III) ions on the impregnated resin.

This better fit of the equilibrium data to the Langmuir isotherm suggests monolayer coverage of Cyanex 272 on Purolite MN 202. Similar results were obtained in the other papers, where the experimental data of the adsorption equilibrium are obtained for Au(III) sorbed on the chemically modified cellulose [26] and N-carboxy methyl chitosan [27] correlated well with the Langmuir isotherm equation.

### 3.6. SEM Studies

**Figure 7:**
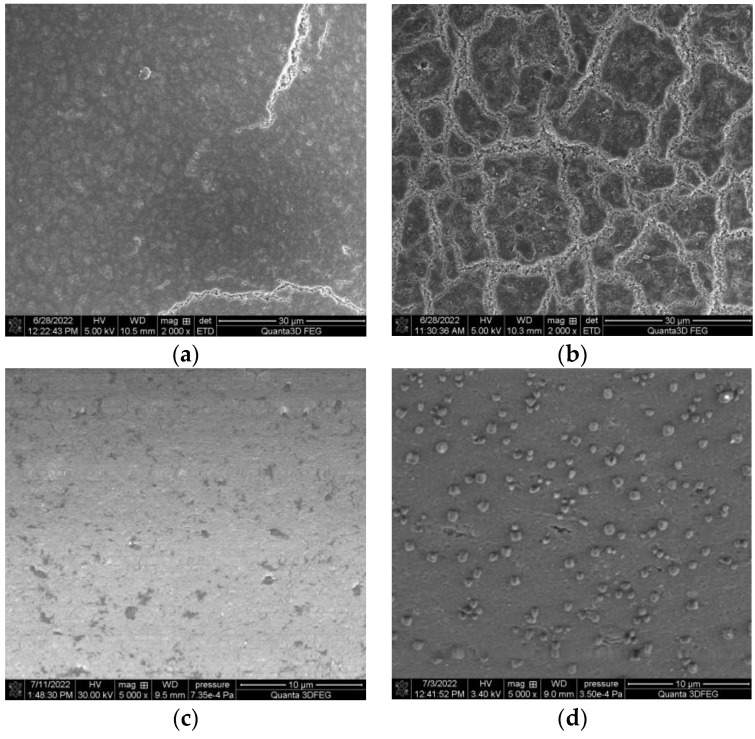
SEM images: (**a**) Purolite MN 202 before the impregnation process; (**b**) Purolite MN 202 after the impregnation process; (**c**) Purolite MN 202 after the sorption of gold(III) ions; (**d**) Purolite MN 202 impregnated with Cyanex 272 after the sorption of gold(III) ions.

The SEM images of Purolite MN 202 before and after the impregnation process are given in Figure 7a,b. The results in Figure 7a,b confirm that Cyanex 272 was present on the Purolite MN 202 surface and the impregnation process was efficient.

The SEM image of Au on Purolite MN 202 is given in Figure 7c. The SEM image of gold shows that the surface of Purolite MN 202 was covered with a thin layer of metallic gold. The SEM image of Au on Purolite MN 202 impregnated with Cyanex 272 is given in Figure 7d. In this case, gold creates small particles on the surface of the impregnated sorbent. The size of particles is about 500 nm. The comparison of two forms of reduced gold on the surface of impregnated and non-impregnated sorbents indicates that the impregnation process affects the form of gold. Formation of gold particles is due to the presence of Cyanex 272 on Purolite MN 202.

### 3.7. Recovery of Gold from the Modular Connector RJ 45 (8P8C)

**Figure 8:**
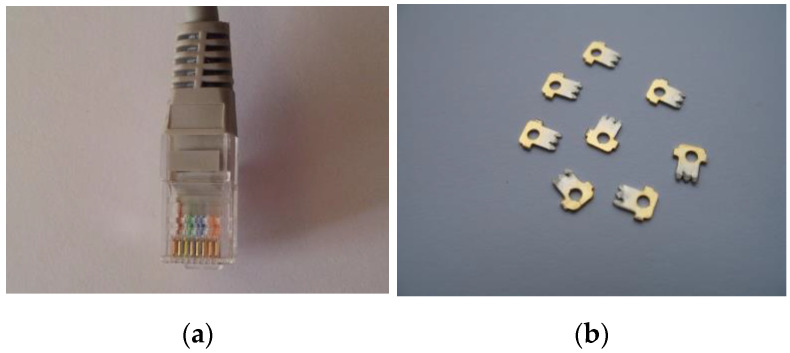
(**a**) Modular connector RJ 45 (8P8C); (**b**) recycled pins.

The pins were recycled from the modular connector RJ 45 (8P8C) (an eight-wire connector used commonly to connect computers (LAN), especially Ethernets) (Figure 8a,b)).

Table 3 presents the composition of the solution after the pin leaching process.

**Figure 9:**
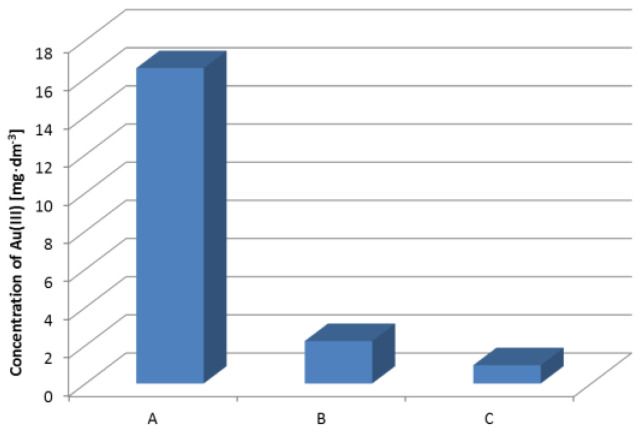
Removal of Au(III) ions from the solution the pin leaching: A—the initial concentration of gold(III) ions. B—the concentration of gold(III) ions after the sorption on Purolite MN 202. C—the concentration of gold(III) ions after the sorption on Purolite MN 202 impregnated with Cyanex 272.

The main components of the solution were copper(II) and nickel(II) ions. The content of gold(III) ions was 16.53 mg·dm^−3^. The colour of the solution was dark green without residues after the digestion process. The hydrochloric acid–hydrogen peroxide system is efficient and does not need the use of nitric(V) acid. There is no emission of toxic nitrogen oxides. Moreover, the solution after leaching does not contain nitrate(V) ions. The sorption of gold(III) ions from the solution after the pin leaching was performed on Purolite MN 202 before and after the impregnation process. The results of the sorption process are presented in Figure 9.

As follows from the results, the concentration of gold(III) after the sorption process was lower for Purolite MN 202 impregnated with Cyanex 272 than for non-impregnated Purolite MN 202. This result confirms that the impregnation process is efficient.

### 3.8. Limitation of Purolite MM 202 Impregnated with Cyanex 272

The impregnated sorbent used in this paper shows good adsorption capacity for gold(III) ions. In acidic solutions after the digestion of connectors, the sorbent is selective and allows for the separation of gold(III) ions. One disadvantage resulting from the presence of Cyanex 272 can be the loss of selectivity in neutral and alkaline solutions. Cyanex 272 can extract copper(II) ions under these conditions.

## 4. Desorption Studies

**Figure 10:**
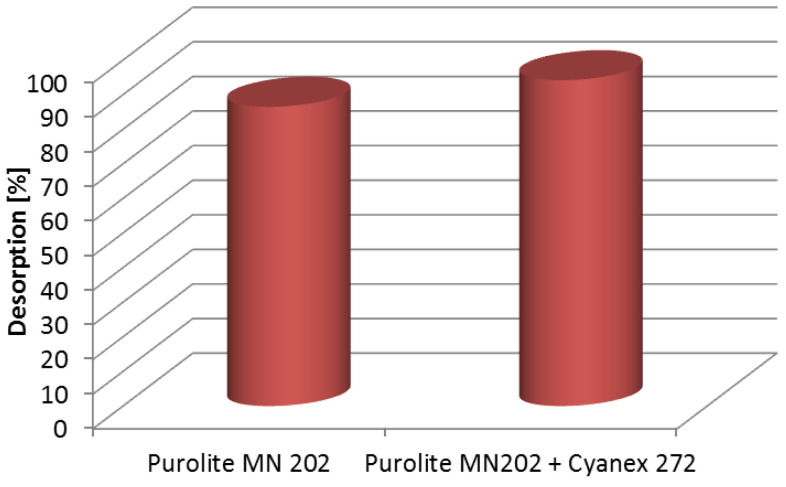
Desorption of gold(III) ions using 5% thiourea in a 0.1 M HCl solution.

The results of the desorption process are presented in Figure 10.

As follows from the results, the desorption process was more efficient for Purolite MN 202 impregnated with Cyanex 272 (94.19%) than for non-impregnated Purolite MN 202 (86.51%). These results demonstrate the effectiveness of using the impregnated sorbent for the sorption and desorption of gold(III) ions. Given the good desorption efficiency of gold(III) ions and the high sorption capacity, it is possible to reuse the impregnated sorbent. However, industrial application can be possible after more extensive studies, including, e.g., 100 sorption-desorption cycles.

## 5. Conclusions

Currently, the amount of e-waste is constantly increasing; therefore, it is a source of valuable metals such as gold.

The results demonstrated that Purolite MN 202 impregnated with Cyanex 272 removes gold(III) ions effectively from modular connectors RJ 45 (8P8C) compared with non-impregnated Purolite MN 202. The results can be applied to gold recovery from e-waste.

The presented method of gold recovery overcomes the problem of emission of nitrogen oxides that can form during the dissolution of e-waste using the aqua regia.

In addition to acidic solutions, cyanides are also used for gold leaching [28]. Cyanide is considered to be a hazardous compound because of its toxicity. The method of gold dissolution and recovery presented in this paper does not require the use of toxic cyanides.

## Figures and Tables

**Table 1:** Kinetic parameters for the unmodified Purolite MN 202 and after the impregnation with Cyanex 272 determined from the results obtained using the static method.

Pseudo Second Order	Ion	HCl Concentration [M]	Kinetic Parameters				
			R^2^	*q*_2_ (mg·g^−1^)	*k*_2_ (g·(mg·min)^−1^)	*h* (mg·(g·min)^−1^)	SD (Standard Deviation)
Before impregnation							
Type 1	Au(III)	0.001	0.99980	9.96	0.0512	5.08	0.18
		0.1	0.99986	9.18	0.3072	25.88	0.16
		1	0.99995	9.28	0.3569	30.71	0.09
		3	0.99998	9.46	0.2378	21.29	0.06
		6	0.99997	9.32	0.1457	12.65	0.08
After impregnation							
Type 1	Au(III)	0.001	0.99974	9.96	0.0088	0.88	0.77
		0.1	0.99970	8.59	0.0271	2.00	0.25
		1	0.99970	9.12	0.0766	6.36	0.18
		3	0.99989	9.47	0.0488	4.37	0.14
		6	0.99980	9.49	0.0324	2.92	0.18

**Table 2:** Adsorption isotherm constants for Purolite MN 202 before and after the impregnation by Cyanex 272 determined from the linear analysis using the Langmuir and Freundlich isotherm models.

Sorbent	HCl Concentration [M]	Langmuir Isotherm Parameters			Freundlich Isotherm Parameters		
		*q*_0_, mg·g^−1^	*b*, dm^3^·mg^−1^	R^2^	*n*	*K_F_*, dm^3^·g^−1^	R^2^
Purolite MN 202 before impregnation	0.001	162.97	0.0588	0.922	1.338	9.997	0.935
	0.1	140.25	0.0429	0.992	1.451	7.437	0.961
	1	53.41	0.0152	0.996	2.355	3.725	0.978
	3	56.38	0.0228	0.997	2.528	5.116	0.970
	6	58.30	0.0145	0.997	2.214	3.524	0.975
Purolite MN 202 after impregnation	0.001	259.45	0.0292	0.983	1.177	7.937	0.992
	0.1	187.03	0.0296	0.999	1.290	6.501	0.990
	1	156.32	0.0051	0.995	1.304	1.444	0.995
	3	167.55	0.0057	0.997	1.284	1.609	0.994
	6	172.33	0.0059	0.996	1.281	1.702	0.995

**Table 3:** Contents of metal ions in the solution after the digestion of the modular connector RJ 45 (8P8C).

Metal Ions/Concentration, mg·dm^−3^					
Au^3+^	Ni^2+^	Co^2+^	Fe^3+^	Cu^2+^	Zn^2+^
16.53	349.6	0.45	7.4	57,400	42.8

## Data Availability

The data are included in the article and available upon request.
